# Supplementary material for: A qualitative, grounded theory exploration of the determinants of self-care behavior among Indian patients with a lived experience of chronic heart failure
Source: PLoS One. 2021 Jan 27;16(1):e0245659. doi: 10.1371/journal.pone.0245659 (PMC7840054; doi:10.1371/journal.pone.0245659)
Supplement: Attachment — Submitted filename: Responses_PLoSOne.docx [file pone.0245659.s001.docx]

**Note to the Academic Editor**:

Dear Editor,

We are very grateful to both reviewers who have made astute observations that have helped improve the quality of this manuscript. We have followed the journal’s guidelines for the requested revisions.

**Reviewer #1**:

**Comment #1)** In General:- Be consistent in used terminology: For example - “South Asian” and Indian are used interchangeably. However, Pakistan, Nepal, Bhutan, Bangladesh, Afghanistan, Sri Lanka and the Maldives are also part of South Asia. It can be argued the results of your research are not applicable to heart failure patients in these countries since :
- these countries have different cultures and thus it is possible HF-patients in these countries meet
other barriers and facilitators to self-care behavior.
- it is very difficult to generalise results of qualitative research.
Therefore it is not clear to me if the results are transferable to other South Asian countries and I suggest you use the "Indian" instead of "South Asian".

**Response #1** – We agree with this important point. Owing to the significant cultural (ethnic, religious, socio-economic) diversity of the South Asian population, the results of this study may not be generalizable to the whole of South Asia. Accordingly, we have changed the terminology from South Asia to India in all relevant places, i.e, the title of the manuscript and in those sections dealing with study methods, results, discussion and conclusion, both in the abstract and the main paper.

**Comment #2)** Please check with author guidelines if standard British English or American English has to be used; please perform a thorough spell check of the entire document.

**Response #2** – Spell check and corrections have been made at relevant places.

**Comment #3)** Title - A Qualitative grounded theory exploration of the determinants of self-care behavior, among South Asian patients with a lived experience of chronic heart failure.

This title suggests all determinants of self-care behaviour are thoroughly discussed within the paper, however this is not entirely the case. The main focus of the paper is the barriers of self-care. Facilitators are briefly discussed and published elsewhere.

**Response #3** – Thank you for clarifying. While selfcare facilitators are published elsewhere, that paper is a ‘research note’, which meant that we could not discuss (in the Discussion section of that manuscript) the impact of facilitators on selfcare, neither could we examine the findings in it’s socio-cultural perspective as we have discussed in this manuscript. Furthermore, we have listed the facilitators in Table 3 and discussed the impact of both barriers and facilitators in this paper. Hence, we do believe that the title is appropriate (with South Asian patients changed to Indian), since all emerging factors affecting selfcare have been discussed in this paper.

**Comment #4)** Furthermore, this title also suggests the research has taken place in the whole of South Asia, while this is not the case. (see also feedback above)

**Response #4** – Changes have been made as advised.

**Comment #5)** - **Abstract** - Last sentence (line 80) it is stated that addressing these determinants may help to improve medication adherence. What about adherence to other self-care advice such as fluid retention or salt?

**Response #5** – I have replaced the word ‘medication’ with ‘treatment’.

**Comment #6)** – **Introduction** - Please provide a reference for the statement on pag. 3, line 99 and 100: "Adherence to treatments is key to reducing the risk of….."

**Response #6** - I have added the relevant reference.

**Comment #7)** - pg. 4, Line 130: "Factors, principally, gender, the geography and society of their up-bringing, would shape patients belief"; I do not understand the use of “would” in this sentence. Do or don't these factors shape patients beliefs?

**Response #7** – We have deleted ‘would’, since these factors do influence selfcare.

**Comment #8)** – **Methods** - Within the methods you describe on the one hand a written consent was obtained from all participants. On the other hand you state in your results (page 19, line 475) rural poor illiterate or semi-literate patients also participated in your research. Therefore I am wondering how you informed these patients? Did you use the same consent form for all patients or was your consent form tailored to the literacy levels of your patients? Or, if it wasn't tailored, how did you inform and obtained consent of these patients. Please state this in your answer.

**Response #8** – Thank you for noting this. We would like to clarify that the same consent form, translated into the respective vernacular language (through a professional translating agency) was used for these patients; we did not have a scaled down version of the form. There was one female patient who could not read the form at all. In her case, we involved the patient’s caregiver who knew how to read the language and explained study details and procedures to the patient and the caregiver and cleared their doubts. The patient’s thumb impression was obtained on the consent form along with the caregiver’s signature, followed by the investigator’s signature.

Other patients who were semi-literate (basic reading and signing ability) were asked to read the form to the best possible extent, if needed with caregiver’s help. The investigator spent a longer duration with these participants to explain study details and clear doubts. These patients then signed the form, followed by the investigator’s signature.

We have added the following sentence to the manuscript, Page 4, Line 144 – “Informed consent forms were translated into vernacular languages by a professional translating agency and approved by the ethics committee. Written informed consent was obtained from all participants, including semi-literate and illiterate participants in accordance with the Indian Council of Medical Research’s (ICMR) National Ethical Guidelines for Biomedical and Health Research involving Human Participants 2017.”

**Comment #9)** – Results - In general I find the results are presented in a complicated fashion: - Titles and subtitles are difficult to distinguish from one another.

**Response #9** – We agree with your comment. However, we have attempted to simplify the format of presentation of results as far as possible. We hope that results presented in Table 2 will give readers a clear snapshot of themes and their supporting codes.

**Comment #10)** - Terminology used in table 2 is not always used in the description of these themes and codes below. An example: Theme 1; Entrenched beliefs: In table 2, the first code of this theme is symptom/disease abstractions. In the description below you use the term causal abstractions. Please check terminology in the whole document and adjust.

**Response #10** – We sincerely apologize for this oversight and thank you for pointing this out. I have made the required corrections.

**Comment #11)** - Furthermore, in the description of themes and codes, new codes such as “trust deficit or denial/normalization who aren’t shown in table 2, are presented. Please integrate them in table 2.

**Response #11** – Thank you, we have integrated these codes and extracts.

**Comment #12)** Pag. 9, line 256: please place the parenthesis between 14 and 63.6% instead of in front of 14.

**Response #12** – Thank you, I have fixed this.

**Comment #13)** - Pag. 15, line 385: “passive receipt of information”. I assume the authors mean “information seeking passively” since this term is used in table 2. Please note there is a difference between reception of information and seeking information. Check which one of these terms it is you mean and be consistent.

**Response #13** – Thank you. Passive receipt of information in more appropriate; since patients exhibiting passivity do not seek information actively, instead they do not seek information at all or they ‘chance’ upon the information. I have made the changes accordingly.

**Comment #14)** - Intermediate factors: you state they modulate the selfcare process. Please provide a reference for this claim.

**Response #14** – This is an empirical finding emerging from the dataset and memo. We have added a section about how these factors affected selfcare in our patient population.

**Added, Page 22, Discussion section, Line 546** – “The ‘intermediate factors’ that we identified, namely, ‘patient expectations’ and ‘hospital/provider hopping’ are novel findings in our study. We have chosen to call these intermediate factors since factors did not immediately shape selfcare behaviour in our participant cohort. However, participants verbally expressed the fact that the thwarting of their expectations in terms of symptom recurrence or a re-hospitalization despite being adherent to treatment, left them feeling doubtful about the efficacy of treatment and they anticipated switching to other treatments, including alternative medications in future. Hospital hopping may lead to a situation which culminates in the patient seeking specialized care at a tertiary care centre. However, since the patient did not have a trusted relationship with any hospital system or a provider, they did not have a clear action plan in the event of symptom exacerbations.”

**Comment #15)** – Discussion - Pg 21, line 536 (and further) authors state that patients who explicitly and spontaneously expressed responsibility for their own health, displayed better overall self-care and adherence. I would like to bring the authors attention to the fact that this is new information. If this is a result of previous research please provide the appropriate reference supporting this claim.

**Response #15** – This information is new and unique to this study. We have made the following addition – Page 22, Line 536, Indeed, “in our participant cohort”, patients who….”.

**Comment #16)** Pg 22, line 561: authors state they see an interesting overlap of three social constructs….. please provide a reference supporting the information considering social constructs.

**Response #16** – These are findings that emerge from the data, hence we have not referenced it.

**Reviewer #2**:

The manuscript addresses an important topic: the self-care behavior of patients, especially focusing on patients in South Asia. The study uses a grounded theory approach to examine determinants of self-care. The treatment adherence is considered a main part of the self-care process. The self-care concept includes aspects of monitoring, maintenance and management of the disease.
The methodical approach is well explained and comprehensible. 22 patients and 17 caregivers were interviewed. Results were classified into 3 main categories that include several sub-categories. The negative determinants and the intermediate factors are the main focus of this article. The negative effects of self-care include entrenched beliefs, passivity, lack of knowledge, negative emotions, financial difficulties, and fatalism.
Intermediate factors are patients’ expectations, and provider hospital hopping.
The findings contribute to the further improvement and support of patients’ self-care by describing the importance of socio-cultural determinants and analyzing those determinants that are specifically important for patients within South Asia.

**Comment #1** - I have one main comment on the paper: Within the discussion, the authors draw an important link and encourage the reader to understand the results from a sociological perspective. However, the link to theories and current studies of medical sociology is missing. E.g. the mentioned passivity, especially of woman (line 532) could related to the cultural role of woman and social structures in India, which were certainly examined in further studies that not necessarily concern patients with HF but also consider other chronic conditions or general behavior patterns. The aspects considered as “socially constructed roots” (line 567-583) are very interesting but should also be linked to existing study results.

**Response #1** – Thank you for identifying this. We have added the following section along with key references, which we hope will help put the results in better perspective.

**Addition – (Discussion Section)** Physical illness – including its experience – and resultant medical care are intricately influenced by social aspects including gender, to the extent that even knowledge and assumptions about illnesses, and broad aspects of physiology as such, are socially constructed(24). For instance, women inevitably experience significantly greater health strains compared to men due to these socially constructed roles around gender (and traditional gender patters in division of household labor), which interact and condition all components of caregiving, normalizing differential experiences between women and men in healthcare (25). Further, it is known that the role of being ‘sick’ is more socially acceptable for women (germinating from gender roles and statuses, and resultant life circumstances for women), which then extends to even associated health-related behaviors and responses (26). Feminine role expectations, which especially include (unpaid) caregiving invariably lead to negative consequences for women's well-being (27). Men's caregiving (and by that token care-receiving) on the other hand is greatly contingent on the characteristics of men's families (and not their social status, employment, or even earning) – because of which women are treated (and are conditioned to treat themselves) as kin-keepers for all their lives, whereas men's roles as kin-keepers depends on the agency of women in the family and on the equations of marriage (28).

The following comments should be understood as suggestions and minor issues:

**Comment #2** - Unfortunately, possible differences between the perception of patients and caregivers are not reported. Maybe the issues addressed in the methodology section: coping with evolving or caring for their own health (line 183, 184) could be elaborated.

**Response #2** – Thank you for this very pertinent comment. Caregiver interviews were conducted to mainly support themes emerging from patient data. However, we did want to add a section on caregiver’s coping with fluctuating conditions and how they care for their own health. We thought the better of it since the Results and Discussion sections were already long and complex. We intend to publish the results from caregiver interviews as a separate brief ‘research note’ at a later date.

**Comment #3** - The first paragraph of section “Results” (line 245-250) could be rephrased for a better understanding, e.g. line 247 – “of these” could not only refer to the patients included in the study could but also refer to the patients that refused and caregiver that were unavailable. In the abstract n=39 (line 57) participants are mentioned. Whereas in line 247 n=30 and line 250, n=7 participants are mentioned.

**Response #3** – Thank you for pointing this out. We made an inadvertent error in reporting screening numbers (27 instead of 25, with 3 refusing consent). I have further clarified how we arrived at n = 39, i.e, 22 patients and 17 caregivers were interviewed. Additions to the manuscript have been made at relevant places.

**Comment #4** - The gender of the caregivers could be reported too. Line 254 only reports female patients.

**Response #4** – Thank you, we have made the following addition –

**Added**: (Results section, para 2, line 262) - 7 (41.2%) caregivers were male caregivers, while 10 (58.8%) were female caregivers.

**Comment #5** - Line 254 reports the mean age of the participants and states the number of female patients in the following half sentence. This change of focus could confuse the reader.

**Response #5** – We have added mean age “of patients” to clarify this.

**Comment #6** - Table 1: It would help to have the total n within the table.

**Response #6** – Thanks for identifying this; I’ve added total N into the table.

**Comment #7** - How was the data within the section “self-care practices” collected? Was it a questionnaire or was the information collected during the interviews? The interview guide only included edema, blood pressure and weight.

**Response #7** – We had a separate brief data collection form to collect demographic and clinical data. It included a questionnaire with items pertaining to basic selfcare practices and dichotomous responses (Yes/No).

**Comment #8** - In the end of the manuscript some gender-related aspects are reported. Maybe it would be of value to add the information already within the table, e.g. report the self-care practices according to the gender.

**Response #8** – We have described this to the best possible extent (given the manuscript length) in the Results section titled, ‘Memo on gender, geographic, societal factors and social discourses shaping self-care’.

**Comment #9** - Since the number of participants is small, the decimal places could be omitted.

**Response #9** – We have adjusted to one decimal point, since the sample size is small.

**Comment #10** - Table 2: Within the table 2 the punctuation marks should be checked; e.g. sometimes literal speeches are not finished.

**Response #10** – I have corrected this to the best of my ability.

**Comment #10** - For me, the aspect “provide or hospital hopping” is rather an expression of the absence of confidence/trust in the provider and therefore the negation of the trust highlighted in the positive points (table 3). Maybe, the difference could be made clearer.

**Response #10** – Thank you for this astute observation. I have added the following in Discussion, Line 565 – “This behaviour, representing a negation of trust, may be contrasted by the previously published finding in our facilitators of selfcare report, where patients from lower socio-economic strata who had a trustworthy relationship with their physician adhered to long term treatments despite financial constraints(12). Vesting trust in the healthcare system and in the treating team is thus a pivotal attribute of patients and families who display optimum selfcare, among Indian patients.”

**Added to manuscript** - Hospital hopping may lead to a situation which culminates in the patient seeking specialized care at a tertiary care centre. However, since the patient did not have a trusted relationship with any hospital system or a provider, they did not have a clear action plan in the event of symptom exacerbations.

I hope my suggestions help to further improve the paper.
