# Supplementary material for: A qualitative, grounded theory exploration of the determinants of self-care behavior among Indian patients with a lived experience of chronic heart failure
Source: PLoS One. 2021 Jan 27;16(1):e0245659. doi: 10.1371/journal.pone.0245659 (PMC7840054; doi:10.1371/journal.pone.0245659)
Supplement: Attachment — Submitted filename: Response to Reviewers round 2.docx [file pone.0245659.s002.docx]

**Reviewer #1**:

**1.** **Abstract**

- line 48 and 50: you still use “South Asian” instead of Indian. Is there a specific reason or is this an oversight?

**Response**: Thanks for your note. This was deliberate since we did indeed want to make the point that there are data which show that treatment adherence in CVD is particularly poor among the South Asian population.

**2.** **Methods**

- You state in line 194 and 195 that the interviews were performed in 7 languages. I am wondering if the principle interviewers (KDY and BKB) familiar with all of these languages? If not, how did you manage this?

**Response**: KDY has reading, speaking and writing proficiency in 4 languages (English, Hindi, Konkani, Kannada) and can understand a fifth language (Malayalam), while BKB is proficient in 3 (English, Telugu and Kannada). Our research nurse (Ms. Immaculate Sheela) is proficient in Tamil and Malayalam. When patients speaking Tamil and Malayalam interviewed patients, Ms. Immaculate would ask questions in the presence of one of the two principle interviewers, who would ask for on the spot translations if needed and direct further probe questions.

- Line 237: please check the spelling of "nvivo" –

**Response**: Thank you for noting and sincere apologies for the error. I have corrected it.

- Line 240 en 244, figure 1: be coherent in the way you identify participants e.g. "patient 002" (line 240) and "patient_002" (line 244) – Sincere apologies for the oversight; I have corrected the same.

**Response**: Apologies, I have corrected this.

**3. Results**

- Line 307, table 2: please use a coherent manner to report the references to quotes. Sometimes references are placed in front of the quote, sometimes at the end of the quote and sometimes there are no references.

**Response**: Thank you, I have made the necessary corrections.

- Line 329: change 'self perception" to 'self-perception"

**Response**: I have corrected this.

- Line 392: you state "Rather patients were passive...." I believe there is a word missing between "rather" and "patients"

**Response**: I have added “…mostly female..”

**4. Discussion**

- line 579: please remove the additional blank space between "constructed" and "For instance..."

**Response**: I have done so.

- Line 583: please remove the additional blank space between "healthcare" and "further".

**Response**: I have done so.

- Line 629; you describe key components of an intervention plan while using the future simple tense. I agree to use this tense when you state you will develop an intervention plan. However I feel the present simple tense is better suited to describe the content of this intervention plan and to use in your enumeration. for example "key components will include (i) a brief questionnaire developed to elict...." in stead of " a brief questionnalire will be developed to elict..."

**Response**: Thanks for this; I have corrected the tense everywhere.

- Line 635; I don't think a health worker is part of your intervention plan. However, a simple screening tool for mood,...." is.

**Response**: The trained health worker is currently playing a central, important role in the intervention plan. The PHQ-9 tool is administered by the health worker and a brief psychological intervention will be delivered along with a referral to psychiatry if needed.

- Line 637: see comment line 629. again future simple is used in (iv) brief psychological interventions will be delivered....". While present simple is more approprioate to use in a enumeration. Therefore (iv) brief pyschological interventions to improve...." are part of this intervention plan.

**Response**: I have made the change.

**Reviewer #2**: Dear authors, Thank you for your corrections and answers to the questions. You answered most of the questions and the manuscript improved very much. But, from my point of view, it needs further improvement.

1. Regarding comment and response Nr. 7: Thank you for clarifying. When you used information that were derived from a questionnaire, you should mention this additional questionnaire within the method section.

**Response**: Thanks very much for noting this. I have added this under the Methods section, Para titled ‘Eligibility Criteria and sampling’, last sentence, “In addition, we captured basic clinical and demographic data and selfcare practices on a data collection form.”

2. Table 2 – columns of the table split the theme sections. So you don’t know if trust deficit and fatalism are new themes.

**Response**: Sorry for this oversight. I have corrected this.

3. The quote “Nobody told me …” line 389 is differently assigned within table 2. In table 2 the quote is under the headline “Passive receipt of information” whereas in line 389 it is assigned to the one-way compliance. Personally, I don’t see the receipt of information (even a passive one) when I don’t get any information as the quote “Nobody told me.” would suggest.

**Response**: Thanks for your note. This referenced patient had a long history of heart failure but said that she had not known that she had a cardiac problem for a long time. When we asked her if she had inquired of the doctors about the reasons for her symptoms/problems or whether she had tried finding it out from other sources, she replied back saying, “No, nobody told me; even the doctor did not tell me”, but while adhering to her medications as prescribed. However, this had come at the cost of many years of poor monitoring practice. In this context, we coded the statement both ways – (i) that the patient was content passively receiving information from her physician (passive receipt of information), and (ii) that she was content passively following basic treatment regimen advice (one-way compliance).

For want of space, we have mentioned this only once under ‘one-way compliance’ in the main text.

4. The spelling of NVivo differs, e.g. line 225, 237 & 61 – please check spelling and consistency

**Response**: I have made the correction.

5. The Article would be easier to read if you didn’t integrate the subtitles, e.g. “entrenched beliefs or notions” (line 309) or “passivity” (line 371), etc., into sentences. A paragraph after the theme would make the affiliation of the Codes to the themes clearer.

**Response**: Thank you for noting this; I have made the correction.

6. The terms in the table and the occurrence in the text should be in the same order, e.g. denial or normalization (line 359) is following the trust deficit in the text. In the table it follows the obstacle self-perception.

**Response**: Sincere apologies for the error; I have corrected the same at relevant places. (Tables 2 and 3)

7. Sometimes, e. g. line 318 and 328 are :: - is that on purpose or a typo?

**Response**: It was a part of transcribing convention that we had followed. However, in the interests of not confusing the readers, we have removed it from the relevant places.

8. Line 355 signs for literal speech are missing.

**Response**: Thank you – have added it.

9. Please use same terms and order in table 2 as in table 3, e.g. knowledge deficit vs. Lack of knowledge.

**Response**: Thank you – have corrected it.

10. Regarding the social-cultural underpinning, it might be worth to have a look at the “Theory of planned behavior” and integrate it into the discussion. This theory relates attitudes, norms and perception into one behavioral framework.

**Response**: I am very grateful for your suggestion. After your suggestion, I did refer to the Theory of Planned Behaviour. There are certainly overlaps between our findings and what the theory proposes. However, we are proposing a theory from our findings which we have called the ‘Extant Cycle Theory’. I have written up the paper as a ‘Brief Report’/ ‘Research Note’ and we will submit the paper around mid-Jan. Briefly, we’re proposing that the extant cycle theory is a type of ‘situation specific’ theory of selfcare in chronic disease and it has already informed the structure and delivery of the intervention package for the 3 centre RCT for which recruitment has just started. However, we shall now also put our findings in the context of the ‘Theory of Planned Behavior’ and evaluate whether changes are needed.
